# Factors associated with weight loss by age among community-dwelling older people

**DOI:** 10.1186/s12877-023-03993-0

**Published:** 2023-05-06

**Authors:** Tomoko Yano, Kayo Godai, Mai Kabayama, Hiroshi Akasaka, Yasushi Takeya, Koichi Yamamoto, Saori Yasumoto, Yukie Masui, Yasumichi Arai, Kazunori Ikebe, Tatsuro Ishizaki, Yasuyuki Gondo, Hiromi Rakugi, Kei Kamide

**Affiliations:** 1 Division of Health Sciences, Osaka University, Graduate School of Medicine, 1-7 Yamada, Suita, Osaka 565-0871 Japan; 2 Department of Geriatric and General Medicine, Osaka University Graduate School of Medicine, Osaka, Japan; 3 Department of Clinical Thanatology and Geriatric Behavioral Sciences, Osaka University Graduate School of Human Sciences, Osaka, Japan; 4 Tokyo Metropolitan Geriatric and Hospital and Institute of Gerontology, Tokyo, Japan; 5 Center of Supercentenarian Medical Research, Keio University School of Medicine, Tokyo, Japan; 6 Department of Prosthodontics and Oral Rehabilitation, Osaka University Graduate School of Medicine, Osaka, Japan

**Keywords:** Weight loss, Community-dwelling older people, Age groups, Factors, Cohort study

## Abstract

**Background:**

Factors associated with weight loss in community-dwelling older people have been reported in several studies, but few studies have examined factors associated with weight loss by age groups. The purpose of this study was to clarify factors associated with weight loss by age in community-dwelling older people through a longitudinal study.

**Methods:**

Participants in the SONIC study (Longitudinal Epidemiological Study of the Elderly) were community-dwelling people aged 70 or older. The participants were divided into two groups: 5% weight loss and maintenance groups, and compared. In addition, we examined factors affecting weight loss by age. The analysis method used was the χ^2^ test, and the t-test was used for comparison of the two groups. Factors associated with 5% weight loss at 3 years were examined using logistic regression analysis with sex, age, married couple, cognitive function, grip strength, and the serum albumin level as explanatory variables.

**Results:**

Of the 1157 subjects, the proportions showing 5% weight loss after 3 years among all subjects, those aged 70 years, 80 years, and 90 years, were 20.5, 13.8, 26.8, and 30.5%, respectively. In logistic regression analysis, factors associated with 5% weight loss at 3 years by age were influenced by BMI of 25 or higher (OR = 1.90, 95%CI = 1.08–3.34, *p* = 0.026), a married couple (OR = 0.49, 95% = 0.28–0.86, *p* = 0.013), serum albumin level below 3.8 g/dL (OR = 10.75, 95% = 1.90–60.73, *p* = 0.007) at age 70, and the grip strength at age 90 (OR = 1.24, 95%CI = 1.02–1.51, *p* = 0.034), respectively.

**Conclusions:**

The results suggest that factors associated with weight loss by age in community-dwelling older people through a longitudinal study differ by age. In the future, this study will be useful to propose effective interventions to prevent factors associated with weight loss by age in community-dwelling older people.

## Background

In Japan, aging of the national population is very fast and the average life expectancy is the longest in the world [1]. Now, it is considered that extension of healthy life expectancy is the most important issue for health promotion in Japan. Therefore, good nutrition and reduction of older people with malnutrition are important health goals in the National Health Promotion Movement for the 21st Century (Health Japan 21) to extend the healthy life expectancy [2]. In other words, it is necessary to prevent older people living in the community from losing weight due to inadequate food intake.

Factors associated with weight loss in the community-dwelling older people have been reported in several studies, including influences of some diseases such as diabetes mellitus (DM), cognitive decline, smoking, loss of a spouse, low education and low income [3–9]. About nutritional status, several previous studies have shown that insufficient calorie and protein intakeor high rate of carbohydrate intake in the meal may result in weight loss [10–12]. Especially, risk factors for weight loss over aged 70 are supposed to be their having diseases and geriatric syndromes, nutritional status, and socioeconomic background [3–12].

We conducted a meta-analysis of longitudinal studies of weight loss and mortality in community-dwelling older people, and reported that the risk of death was 1.69 times higher in subjects with weight loss than in subjects with maintained bodyweight [13]. This meta-analysis did not reveal weight loss at different age groups. Also, we have reported factors associated with cognitive function decline among different age groups in community-dwelling older people, focusing on blood pressure control [14, 15]. Based on the results of our previous studies, we hypothesized that different factors may contribute to weight loss in different age groups among community-dwelling older people. We considered that factors associated with weight loss differ by age in community-dwelling older people were very important to propose the manners for the preventive care. However, few studies have investigated factors associated with weight loss by age group.

The purpose of this study was to clarify factors associated with weight loss by age in community-dwelling older people through a longitudinal study. In the future, this study will be useful to propose effective interventions to prevent factors associated with weight loss by age in older people.

## Methods

### Study participants

**Fig. 1:**
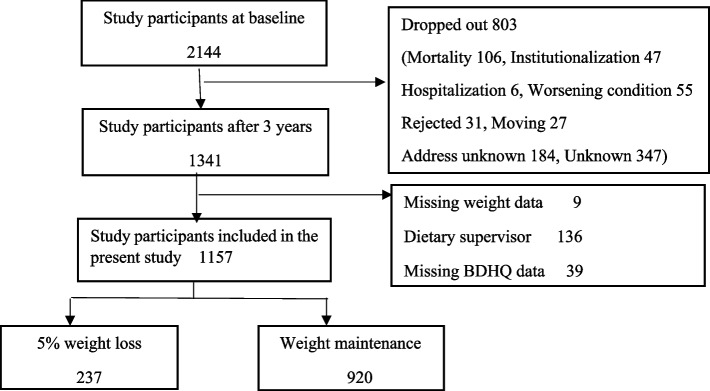
Participants included in the study. Abbreviations: BDHQ, Brief-type self-administered diet history questionnaire

This study analyzed data from the SONIC study (Septuagenarians, Octogenarians, Nonagenarians, and Investigation with Centenaries), a longitudinal cohort survey of community-dwelling older people in Japan [16]. The study began in 2010 with a three-year follow-up survey of community-dwelling older people in four locations in Japan's Kansai and Kanto regions. The study recruited 2144 randomly selected participants in the baseline years of 2011, 2012, and 2013, involving 900 people aged 70–73, 972 people aged 80–81, and 272 people aged 90–91, respectively. Of these, 1341 were participants in the 3-year follow-up survey: 657 people aged 73–76, 610 people aged 83–84, and 74 people aged 92–94, respectively. This study excluded those receiving dietary guidance, those with missing weight measurements, and those with missing BDHQ (brief-type self-administered diet history questionnaire) [17]. Figure 1 shows a flow chart of the study participants. The SONIC study protocol was approved by the institutional review boards of Osaka University Graduate School of Medicine, Dentistry, and Human Sciences, and the Tokyo Metropolitan Institute of Gerontology (approval numbers: 266, H22-E9, 22 018, and 38, respectively). Informed consent was obtained from all participants.

### Weight assessment

In this study, those who lost 5% of their bodyweight from the baseline weight during 3-years follow-up weight were defined as weight losers, using multi frequency body composition scale (Model MC-780A; TANITA Ltd.., Tokyo, Japan) by nurses. In a meta-analysis of weight loss and life expectancy in community-dwelling older people, most studies evaluated 5% weight loss and there was a significant correlation with death in subjects with 5% weight loss over several years [13]. Therefore, 5% weight loss was defined as such in this study. Weight was classified as 5% weight loss or maintenance. We attempted to clarify unintentional weight loss without any dietary restrictions or excessive exercise. This study excludes those who are undergoing weight loss or dietary guidance to improve obesity or metabolic syndrome suggesting intended weight loss according to information in BDHQ questionnaire.

### Health status

This survey was conducted by a physician or nurse using a questionnaire that included physical factors, medical history, and prescribed medications. Blood pressure measurements, body measurements, and blood draws were done by a doctor or nurse [14, 15]. BMI was calculated from weight and height measurements. Serum albumin, total protein, blood glucose, and HbA1C were from blood data. There were several studies indicating that low level of serum albumin was good indicator for malnutrition [10, 18–20]. Furthermore, serum albumin level below 3.8 g/dl is thought to be cutoff for malnutrition [10, 18]. Therefore, we used this criterion for the malnutritional state in the present study. Hypertension was defined as a systolic blood pressure of 140 mmHg or higher, a diastolic blood pressure of 90 mmHg or higher, and the use of antihypertensive medication, according to the Japanese Society of Hypertension guidelines 2019 [21]. DM was defined by the Japanese Diabetes Society as fasting blood glucose of 126 mg/dL or higher, blood glucose of 200 mg/dL or higher at any time, hemoglobin A1C of 6.5% or higher, and the use of diabetic medication [22]. Smoking and drinking histories were categorized into two: current smoking and no smoking or current drinking and no drinking. The grip strength was measured using a Smedley grip strength meter (Model YD-100; Yagami Ltd.., Tokyo, Japan), and the average of two measurements was used.

### Dietary assessment

Dietary intake was assessed using the brief-type self-administered diet history questionnaire. BDHQ was assessed meals eaten during the past month, approximately 100 nutrient intakes and 58–67 food intakes are calculated, in addition to energy and water [17]. Also, BDHQ is calculated by standardizing the amount of physical activity. In this study, of the BDHQ used energy intake, and the energy ratio (% energy) of carbohydrate, protein, animal protein, vegetable protein, and fat. We also classified energy intake and the energy ratio (% energy) of carbohydrate, protein, animal protein, vegetable protein, and fat using the energy ratios in the Dietary Intake Standards for Japanese (2020 version): carbohydrates were classified as less than 50%, 50–65%, and 65% or more, protein as less than 15%, 15–20%, and 20% or more, and lipids as less than 20%, 20–30%, and 30%or more [23].

### Other factors

The survey included social factors such as living arrangement, economic status, and years of education. For living arrangement, living alone, a married couple, and with other were used. The economic status was based on household income, with the options of no financial comfort, normal, and financial comfort. Years of education were defined as 9 years or fewer, 10–12 years, and 13 years or more.

Cognitive function was assessed using Moca-J (The Japanese version of the Montreal Cognitive Assessment), which was developed as a screening test to detect mild cognitive impairment (MCI) [24]. MOCA-J was developed as a screening test to detect mild cognitive impairment (MCI). MCI is suspected if the score is below 25 points.

### Statistical analysis

All the variables have been measured at baseline. Descriptive statistics are summarized as the mean ± SD or median (IQR) for continuous variables and percentages for categorical variables. The continuous variables were checked the normal distribution by visual inspection and the Kolmogorov–Smirnov test. We used the chi-square test for categorical variables, the t-test for continuous variables, and the Mann–Whitney U test for comparison between the two groups. Cochran-Armitage trend tests were conducted. Both univariate and multivariate logistic regression analyses were performed in the present study. In multivariate logistic regression analyses, same adjusted variables were used in all performed analyses. These variables including sex, BMI, living alone, married couples, cognitive function, grip strength, serum albumin level, serum total protein level, protein energy ratio and carbohydrate energy ratio were factors that have been suggested to be associated with weight loss in previous studies and that were significant factors in a single regression in the present study [3–12]. All data were analyzed using the statistical software SPSS Ver. 25 (IBM Japan, Tokyo, Japan). The significance level was set at less than 5%.

## Results

As a result of the longitudinal analysis of factors associated with weight loss by age among community-dwelling older people, 1157 subjects were included in the analysis of this study after excluding 9 subjects with weight data deficit, 136 subjects receiving dietary guidance, and 39 subjects with BDHQ survey deficit (Fig. 1).

**Fig. 2:**
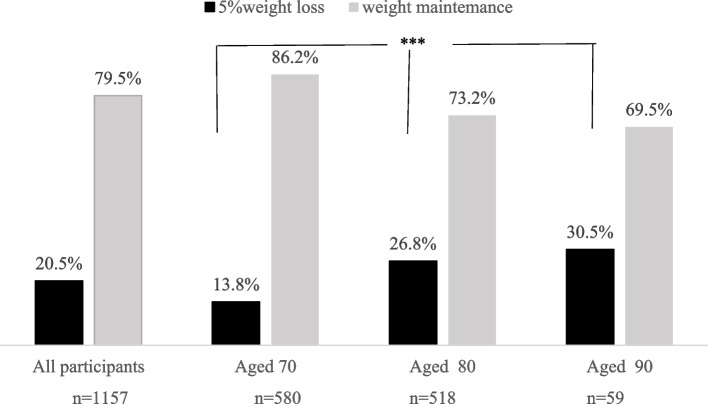
Percentage of 5% weight loss and weight maintenance after 3 years. *P*-values from Cochran-Armitage trend tests; *** *P*-values < . 0001

Of all subjects, 580 people were aged 70 (50.1%), 518 people were aged 80 (44.8%), and 59 people were aged 90 (5.1%). Those who lost 5% of their bodyweight after 3 years comprised 237 people (20.5%), 80 people aged 70 (13.8%), 139 people aged 80 (26.8%), and 18 people aged 90 (30.5%), respectively (Fig. 2). The Cochran-Armitage trend showed a significant increase in the number with 5% weight loss with increasing age (p < 0.001). Subjects with baseline BMI below 18.5, defined as critical low bodyweight numbered 87 people (7.5%), 33 people aged 70 (5.7%), 44 people aged 80 (8.5%), and 10 people aged 90 (16.9%), respectively.

Compared with those with 5% weight loss and maintenance, all subjects were significantly different or associated with a higher mean age (p < 0.001), less married-couple (*p* = 0.06), lower Moca-J scores (p < 0.001), weaker grip strength (*p* = 0.002) (Table 1).

**Table 1:** Comparison of baseline characteristics of 5% weight loss and weight maintenance among all participants

	All participants *n* = 1157	5% weight loss *n* = 237	weight maintenance *n* = 920	*P-*value
Female n(%)	604(52.2)	130(54.9)	446(48.5)	.382^a^
Age M(SD)	76.9( 4.6)	78.4( 4.7)	76.6( 4.5)	< .001^b^
Aged 70 n(%)	580(50.1)	80(33.8)	500(54.3)	< .001^a^
Aged 80 n(%)	518(44.8)	139(58.5)	379(41.2)	
Aged 90 n(%)	59( 5.1)	18( 7.6)	41( 4.5)	
Bodyweight(kg) M(SD)	55.3( 9.8)	55.2(10.2)	55.3( 9.7)	.833^b^
BMI < 18.5 n(%)	87( 7.5)	15( 6.4)	72( 7.8)	.657^a^
18.5–24.9	841(72.7)	170(72.3)	671(72.8)	
≧25.0	229(19.8)	50(21.3)	179(19.4)	
Living arrangemen n(%)				
living alone	219(19.0)	44(17.4)	178(19.4)	.046^a^
married couple	478(41.4)	85(36.0)	393(42.8)	
with other	457(39.6)	110(46.6)	347(37.8)	
Financial n(%) *n* = 1156				
no comfort	225(19.5)	44(18.6)	181(19.7)	.886^a^
normal	682(59.0)	143(60.3)	539(58.7)	
comfortable	249(24.0)	50(21.1)	199(21.7)	
Education n(%) *n* = 1155				
≦9 years	276(23.9)	62(26.2)	214(23.3)	.066^a^
10–12 years	540(46.8)	95(40.1)	445(48.5)	
≧13 years	339(29.4)	80(33.8)	259(28.2)	
Currently smoking n(%)	103( 9.1)	13( 5.6)	90(10.0)	.040^a^
Currently drinking n(%)	415(36.8)	82(35.5)	333(37.1)	.702^a^
Moca-J score M(SD)	23.2( 3.6)	22.4( 3.5)	23.4( 3.6)	< .001^b^
Grip strength M(SD)	24.0( 8.1)	22.5( 8.1)	24.3( 8.1)	.002^b^
Hypertension n(%)	830(79.6)	185(82.2)	645(68.0)	.304^a^
Diabetes mellitus n(%)	155(16.8)	34(16.7)	121(16.8)	.969^a^
Cancer n(%)	156(13.6)	29(12.3)	127(13.9)	.504^a^
Joint diseases n(%)	421(36.7)	82(35.0)	339(37.1)	.595^a^
Disease count M(SD)	3.9(2.4)	3.7(2.2)	3.9(2.4)	.174^b^
Blood glucose(g/dl) M(SD)	109.8(33.8)	110.3(36.9)	109.6(32.9)	.794^b^
HbA1c % M(SD)	5.7( 0.6)	5.6( 0.5)	5.6( 0.7)	.584^b^
Serum albumin n(%) *n* = 1137				
< 3.8 g/dL	17( 1.5)	6( 2.6)	11( 1.2)	.189^a^
3.8–4.1 g/dL	234(20.6)	42(17.9)	192(21.3)	
≧4. 1 g/dL	886(77.9)	186(79.5)	700(77.5)	
Total protein n (%) *n* = 1137				
< 6.5 g/dL	15( 1.3)	6( 2.6)	9( 1.0)	.008^a^
6.5–8.0 g/dL	1065(93.7)	209(89.3)	856(94.8)	
≧8.0 g/dL	57( 5.0)	19( 8.1)	38( 4.2)	
Energy intake kcal M(SD)	1969.7(608.3)	2036.6(640.1)	1962.6(599.1)	.059^b^
Energy intake Female n(%)				
< 1400 kcal	136(22.5)	24(18.5)	112(23.6)	.317^a^
1400–1650 kcal	139(23.0)	28(21.5)	111(23.4)	
≧1650 kcal	329(54.8)	78(60.0)	251(53.0)	
Energy intake Male n (%)				
< 1800 kcal	167(30.3)	25(23.6)	142(31.8)	.229^a^
1800–2100 kcal	121(21.9)	27(25.5)	94(21.1)	
≧2100 kcal	264(47.8)	54(50.9)	210(47.1)	
Carbohydrates %energy n(%)				
< 50%energy	350(30.3)	58(24.5)	292(31.7)	.018^a^
50–65%energy	719(62.1)	153(64.6)	566(61.5)	
≧65%energy	88( 7.6)	26(11.0)	62( 6.7)	
Protein %energy n(%)				
< 15%energy	406(35.1)	89(37.6)	317(34.5)	.606^a^
15–20%energy	594(51.3)	479(52.1)	115(48.5)	
≧20%energy	157(13.6)	33(13.9)	124(13.5)	
Animal protein %energy M(SD)	9.7( 3.4)	9.5( 3.4)	9.7( 3.4)	.490^b^
Plant protein %energy M(SD)	6.7( 1.1)	6.8( 1.2)	6.7( 1.1)	.233^b^
Fat %energy n(%)				
< 20%energy	164(14.2)	38(16.0)	126(13.7)	.125^a^
20–30%energy	738(63.8)	158(66.7)	580(63.0)	
≧30%energy	255(22.0)	41(17.3)	214(23.3)	
Animal fat%energy M(SD)	12.3( 3.8)	11.8( 3.9)	12.4( 3.8)	.030^b^
Plant fat %energy M(SD)	13.5( 3.5)	13.3( 3.4)	13.6( 3.5)	.275^b^

*BMI* Body Mass Index, *Moca-J* The Japanese version of the Montreal Cognitive Assessment, *M* Mean, *SD* Standard deviation

^a^*P-*values from chi-square test

^b^*P-*values from Fisher’s exact test for categorical variables and independent t-test for continuous

Regarding analysis in each age group, they were significantly different or associated with a higher percentage of BMI > 25 (*p* = 0.025), fewer married couple (*p* = 0.005), greater grip strength (*p* = 0.003), higher percentage of serum albumin less than 3.8 g/dL (*p* = 0.004), serum total protein below 6.5–8.0 g/dL (*p* = 0.024), at the age of 70 (Table 2).

**Table 2:** Comparison of baseline characteristics of 5% weight loss and weight maintenance at age 70

	All participants *n* = 580	5% weight loss *n* = 80	weight maintenance *n* = 500	*P-*value
Female n(%)	309(53.3)	37(46.3)	272(54.4)	.186^a^
Bodyweight(kg) M(SD)	56.9( 9.7)	60.0(10.6)	56.4( 9.5)	.002^b^
BMI < 18.5 n(%)	33( 5.7)	4(5.1)	29( 5.8)	.025^a^
18.5–24.9	422(72.8)	48(61.5)	374(74.5)	
≧25.0	125(21.6)	26(33.3)	99(19.7)	
Living arrangemen n(%)				
living alone	90(15.5)	8(10.0)	82(16.4)	.005^a^
married couple	252(43.5)	26(32.5)	226(45.3)	
with other	237(40.9)	46(57.5)	191(38.3)	
Financial n(%) *n* = 579				
no comfort	129(22.3)	18(22.5)	111(22.2)	.883^a^
normal	345(59.6)	46(57.5)	299(59.9)	
comfortable	105(18.1)	16(20.0)	89(17.8)	
Education n(%) *n* = 578				
≦9 years	113(19.6)	17(21.3)	96(19.3)	.825^a^
10–12 years	300(51.9)	39(48.8)	261(52.4)	
≧13 years	165(28.5)	24(30.0)	141(28.3)	
Currently smoking n(%)	85(14.9)	8(10.0)	77(15.7)	.236^a^
Currently drinking n(%)	234(40.3)	36(45.0)	198(40.2)	.463^a^
Moca-J score M(SD)	24.2( 3.1)	23.9( 3.0)	24.3( 3.1)	.302^b^
Grip strength M(SD)	25.8( 8.4)	26.6( 8.6)	25.8( 8.4)	.002^b^
Hypertension n(%)	349(60.2)	53(66.3)	296(59.2)	.657^a^
Diabetes mellitus n(%)	61(10.5)	7( 8.8)	54(10.8)	.687^a^
Cancer n(%)	73(12.7)	7( 8.8)	66(13.2)	.362^a^
Joint diseases n(%)	230(39.9)	31(39.2)	199(40.0)	.903^a^
Disease count M(SD)	4.5(2.6)	4.6(2.9)	4.4(2.6)	.750^b^
Blood glucose(g/dl) M(SD)	112.7(32.6)	112.3(31.3)	112.8(32.8)	.897^b^
HbA1c % M(SD)	5.8( 0.6)	5.7( 0.5)	5.8( 0.6)	.398^b^
Serum albumin n(%) *n* = 562				
< 3.8 g/dL	7( 1.2)	4( 5.1)	3( 0.6)	.004^a^
3.8–4.1 g/dL	113(20.1)	14(17.9)	99(20.5)	
≧4. 1 g/dL	442(78.6)	60(76.9)	382(77.5)	
Total protein n (%) *n* = 562				
< 6.5 g/dL	8( 1.4)	2( 2.6)	6( 1.2)	.024^a^
6.5–8.0 g/dL	528(94.0)	68(87.2)	460(95.0)	
≧8.0 g/dL	26( 4.6)	8(10.3)	18( 3.7)	
Energy intake kcal M(SD)	1958.3(594.9)	2032.1(674.9)	1946.5(580.9)	.232^b^
Carbohydrates %energy n(%)				
< 50%energy	207(35.7)	21(26.3)	186(37.2)	.163^a^
50–65%energy	343(59.1)	54(67.5)	289(57.8)	
≧65%energy	30( 5.2)	5( 6.3)	25( 5.0)	
Protein %energy n(%)				
< 15%energy	205(35.3)	39(48.8)	166(33.2)	.025^a^
15–20%energy	295(50.9)	33(41.3)	262(52.4)	
≧20%energy	80(13.8)	8(10.0)	72(14.4)	
Animal protein %energy M(SD)	9.8( 3.3)	9.1( 3.2)	9.9( 3.4)	.036^b^
Plant protein %energy M(SD)	6.6( 1.1)	6.6( 1.2)	6.7( 1.1)	.426^b^
Fat %energy n(%)				
< 20%energy	67(11.6)	9(11.3)	58(11.6)	.096^a^
20–30%energy	380(65.6)	60(75.0)	320(64.0)	
≧30%energy	133(22.9)	11(13.8)	122(24.4)	
Animal fat%energy M(SD)	12.6( 3.8)	11.9( 3.9)	12.7( 3.7)	.093^b^
Plant fat %energy M(SD)	13.7( 3.5)	13.3( 3.3)	13.7( 3.6)	.327^b^

*BMI* Body Mass Index, *Moca-J* The Japanese version of the Montreal Cognitive Assessment, *M* Mean, *SD* Standard deviation

^a^*P-*values from chi-square test

^b^*P-*values from Fisher’s exact test for categorical variables and independent t-test for continuous

At the age of 80, there was a significant difference or association between weaker grip strength (*p* = 0.002) (Table 3).

**Table 3:** Comparison of baseline characteristics of 5% weight loss and weight maintenance at age 80

	All participants *n* = 518	5% weight loss *n* = 139	weight maintenance *n* = 379	*P-*value
Female n(%)	261(50.4)	81(59.7)	178(47.0)	.013^a^
Bodyweight(kg) M(SD)	54.1( 9.5)	52.7( 8.8)	54.6( 9.6)	.034^b^
BMI < 18.5 n(%)	44( 8.5)	9( 6.5)	35( 9.2)	.216^a^
18.5–24.9	377(72.8)	109(78.4)	268(70.7)	
≧25.0	97(18.7)	21(15.1)	76(20.1)	
Living arrangemen n(%)				
living alone	108(20.9)	29(21.0)	79(20.9)	.853^a^
married couple	219(42.4)	56(40.6)	163(43.1)	
with other	189(36.6)	53(38.4)	136(36.0)	
Financial n(%)				
no comfort	85(16.4)	23(16.5)	62(16.4)	.915^a^
normal	307(59.3)	84(60.4)	223(58.8)	
comfortable	126(24.3)	32(23.0)	94(24.8)	
Education n(%)				
≦9 years	141(27.2)	39(28.1)	102(26.9)	.355^a^
10–12 years	215(41.5)	51(36.7)	164(43.3)	
≧13 years	162(31.3)	49(35.3)	113(29.8)	
Currently smoking n(%)	17( 3.3)	4( 2.9)	13( 3.5)	.745^a^
Currently drinking n(%)	170(33.7)	41(30.4)	129(34.9)	.395^a^
Moca-J score M(SD)	22.3( 3.6)	21.9( 3.4)	22.5( 3.7)	.088^b^
Grip strength M(SD)	22.4( 7.3)	20.7( 7.1)	23.0( 7.3)	.002^b^
Hypertension n(%)	436(84.2)	118(84.9)	318(83.9)	.784^a^
Diabetes mellitus n(%)	81(16.5)	24(17.6)	57(16.0)	.687^a^
Cancer n(%)	71(13.8)	18(12.9)	53(14.1)	.775^a^
Joint diseases n(%)	166(32.5)	44(32.1)	122(32.6)	.914^a^
Disease count M(SD)	3.1(1.8)	3.1(1.8)	3.1(1.7)	.953^b^
Blood glucose(g/dl) M(SD)	105.5(33.2)	106.8(39.8)	105.0(30.5)	.585^b^
HbA1c % M(SD)	5.5( 0.6)	5.6( 0.9)	5.5( 0.5)	.164^b^
Serum albumin n(%) *n* = 526				
< 3.8 g/dL	6( 1.7)	1( 0.7)	5( 1.3)	.592^a^
3.8–4.1 g/dL	106(20.5)	25(18.4)	81(21.4)	
≧4. 1 g/dL	404(78.3)	112(81.2)	292(77.2)	
Total protein n (%) *n* = 526				
< 6.5 g/dL	6( 1.2)	3( 2.2)	3( 0.8)	.261^a^
6.5–8.0 g/dL	481(93.2)	125(90.6)	356(94.2)	
≧8.0 g/dL	29( 5.6)	10( 7.2)	19( 5.0)	
Energy intake kcal M(SD)	1981.9(617.6)	2033.1(632.0)	1963.1(612.0)	.253^b^
Carbohydrates %energy n(%)				
< 50%energy	124(24.0)	29(20.9)	95(25.1)	.112^a^
50–65%energy	342(66.0)	90(64.7)	252(66.5)	
≧65%energy	52(10.0)	20(14.4)	32( 8.4)	
Protein %energy n(%)				
< 15%energy	188(36.3)	49(35.3)	139(36.7)	.830^a^
15–20%energy	263(50.8)	70(50.4)	193(50.9)	
≧20%energy	67(12.9)	20(14.4)	47(12.4)	
Animal protein %energy M(SD)	9.5( 3.4)	9.5( 3.5)	9.5( 3.3)	.806^b^
Plant protein %energy M(SD)	6.8( 1.1)	7.0( 1.2)	6.8( 1.1)	.080^b^
Fat %energy n(%)				
< 20%energy	91(17.6)	27(19.4)	64(16.9)	.563^a^
20–30%energy	323(62.4)	88(63.3)	235(62.0)	
≧30%energy	104(20.1)	24(17.3)	80(21.1)	
Animal fat%energy M(SD)	11.8( 3.8)	11.4( 3.8)	12.0( 3.8)	.122^b^
Plant fat %energy M(SD)	13.3( 3.5)	13.3( 3.3)	13.3( 3.5)	.233^b^

*BMI* Body Mass Index, *Moca-J* The Japanese version of the Montreal Cognitive Assessment, M, Mean, *SD* Standard deviation

^a^*P-*values from chi-square test

^b^*P-*values from Fisher’s exact test for categorical variables and independent t-test for continuous

However, at 90 years, no significant difference or association was found among underweight individuals (Table 4), and no significant difference or association was found among current smoking, current drinking, hypertension, DM, cancer and other diseases, blood glucose, HbA1C, calories, and lipid energy ratio in each age group.

**Table 4:** Comparison of baseline characteristics of 5% weight loss and weight maintenance at age 90

	All participants *n* = 59	5% weight loss *n* = 18	weight maintenance *n* = 41	*P-*value
Female n(%)	34(57.6)	10(55.6)	24(58.5)	.831^a^
Bodyweight(kg) MD(IQR)	49.4(43.0–58.0)	51.7(47.0–59.0)	48.3(41.7–57.5)	.100^b^
BMI < 18.5 n(%)	10(16.9)	2(11.1)	8(19.5)	.598^a^
18.5–24.9	42(71.2)	13(72.2)	29(70.7)	
≧25.0	7(11.9)	3(16.7)	4( 9.8)	
Living arrangemen n(%)				
living alone	21(35.6)	4(22.2)	17(41.5)	.338^a^
married couple	7(11.9)	3(16.7)	4( 9.8)	
with other	31(52.5)	11(61.1)	20(48.8)	
Financial n(%)				
no comfort	11(18.6)	3(16.7)	8(19.5)	.062^a^
normal	30(50.8)	13(72.2)	17(41.5)	
comfortable	18(30.5)	2(11.1)	16(39.0)	
Education n(%)				
≦9 years	22(37.3)	6(33.3)	16(39.0)	.055^a^
10–12 years	25(42.4)	5(27.8)	20(48.8)	
≧13 years	12(20.3)	7(38.9)	5(12.2)	
Currently smoking n(%)	1( 3.3)	1( 5.3)	0( 0.0)	.308^a^
Currently drinking n(%)	11(21.6)	5(31.3)	6(17.1)	.288^a^
Moca-J score MD(IQR)	20.0(18.0–23.0)	20.0(19.0–22.0)	20.0(18.0–23.0)	.812^b^
Grip strength MD(IQR)	18.3(13.4–22.8)	20.0(18.0–23.0)	18.0(13.0–22.3)	.201^b^
Hypertension n(%)	55(81.8)	14(77.8)	31(83.8)	.713^a^
Diabetes mellitus n(%)	13(22.4)	3(17.6)	10(24.4)	.736^a^
Cancer n(%)	12(20.3)	4(22.2)	8(19.5)	.812^a^
Joint diseases n(%)	25(42.4)	7(38.9)	18(43.9)	.340^a^
Disease count MD(IQR)	4.0(3.0–7.0)	4.0(3.0–6.0)	5.0(3.0–7.0)	.812^b^
Blood glucose(g/dl) MD(IQR)	112.0(94.0–143.0)	135.0(102.0–144.0)	102.0(93.0–126.0)	.074^b^
HbA1c % MD(IQR)	5.6(5.3–5.9)	5.5(5.2–5.8)	5.6(5.4–6.0)	.452^b^
Serum albumin n(%)				
< 3.8 g/dL	4( 6.8)	1( 5.6)	3( 7.3)	.544^a^
3.8–4.1 g/dL	15(25.4)	3(16.7)	12(29.3)	
≧4. 1 g/dL	40(67.8)	14(77.8)	26(63.4)	
Total protein n (%)				
< 6.5 g/dL	1( 1.7)	1( 5.6)	0( 0.0)	.255^a^
6.5–8.0 g/dL	56(94.9)	16(88.9)	40(97.6)	
≧8.0 g/dL	2( 3.4)	1( 5.6)	1( 2.4)	
Energy intake kcal MD(IQR)	1953.8(1436.7–2388.7)	1829.5(1631.1–2232.8)	1570.1(1394.4–2537.2)	.374^b^
Carbohydrates %energy n(%)				
< 50%energy	19(32.2)	8(44.4)	11(28.9)	.366^a^
50–65%energy	34(57.6)	9(50.0)	25(61.0)	
≧65%energy	6(10.2)	1( 5.6)	5(12.2)	
Protein %energy n(%)				
< 15%energy	13(22.0)	1( 5.6)	12(29.3)	.077^a^
15–20%energy	36(61.0)	12(66.7)	24(58.5)	
≧20%energy	10(16.9)	5(27.8)	5(12.2)	
Animal protein%energy MD(IQR)	9.7(8.1–11.8)	11.3(9.5–14.0)	9.7(7.7–10.9)	.058^b^
Plant protein %energy MD(IQR)	6.5(5.9- 7.3)	6.5(6.0–7.4)	6.4(7.7–10.9)	.633^b^
Fat %energy n(%)				
< 20%energy	6(10.2)	2(11.1)	4( 9.8)	.927^a^
20–30%energy	35(59.3)	10(55.6)	25(61.0)	
≧30%energy	18(30.5)	6(33.3)	12(29.3)	
Animal fat%energy MD(IQR)	13.5(10.0–16.6)	14.6(12.3–17.3)	13.0(9.5–15.7)	.118^b^
Plant fat %energy MD(IQR)	14.4(11.6–16.8)	12.4(10.4–16.4)	14.7(12.0–16.8)	.223^b^

*BMI* Body Mass Index, *Moca-J* The Japanese version of the Montreal Cognitive Assessment, *MD* Median, *IQR* Interquartile range

^a^*P-*values from chi-square test

^b^*P-*values from Fisher’s exact test for categorical variables and the Mann–Whitney U test for continuous

In the multiple logistic regression analysis, factors associated with 5% weight loss after 3 years by age were significantly correlated at age 70, BMI less than 25 compared with BMI more than 25 (OR = 1.90, 95%CI = 1.08–3.34, *p* = 0.026), married-couple compared with no married-couple (OR = 0.49, 95% = 0.28–0.86, *p* = 0.013), and serum albumin level less than 3.8 g/dL compared with more than 3.8 g/dL (OR = 10.75, 95% = 1.90–60.73, *p* = 0.007) (Table 5). Grip strength was affected at age 90(OR = 1.24, 95%CI = 1.02–1.51, *p* = 0.034), but there was no associated factor at age 80. At age 70, baseline BMI more than 25 and serum albumin less than 3.8 may result significantly in 5% weight loss after 3 years, but married couples was significantly associated with maintenance of body weight after 3 years. At age 90, baseline higher grip strength may result in weight loss after 3 years. In other words, 5% weight loss after 3 years was influenced by different factors at ages 70 and 90.

**Table 5:** Factors associated with 5% weight loss at age 70, 80, and 90 years after 3 years

Explanatory variables	Univariable odds ratio^a^ (95% Confidence)	*P-*value	Adjusted odds ratio^b^ (95% Confidence)	*P-*value
All participants				
Female (ref. male)	1.14(0.86–1.52)	.360	1.01(0.64–1.61)	.958
Age	1.08(1.05–1.11)	< .001	1.07(1.03–1.11)	< .001
BMI < 18.5 (ref. > 18.5)	1.24(0.70–2.20)	.468	0.60(0.32–1.12)	.109
BMI > 25.0 (ref. < 25.0)	1.14(0.81–1.62)	.455	1.18(0.82–1.72)	.374
Living alone (ref. married couple)	0.87(0.60–1.27)	.481	0.67(0.44–1.03)	.066
Married couples(ref. no married couples)	0.75(0.56–1.01)	.059	0.78(0.55–1.09)	.139
Moca-J score	0.93(0.90–0.97)	.001	0.96(0.92–1.01)	.091
Grip strength	0.97(0.96–0.99)	.003	0.99(0.96–1.01)	.312
Serum albumin < 3.8 g/dL(ref. > 3.8 g/dL)	2.13(0.78–5.83)	.139	1.80(0.62–5.14)	.287
Total protein < 6.5 g/dL (ref. > 6.5 g/dL)	2.61(0.92–7.42)	.071	2.32(0.92–6.88)	.070
Carbohydrates > 65%energy(ref. < 65%)	1.71(1.05–2.76)	.030	1.33(0.76–2.32)	.316
Protein < 15%energy (ref. > 15%)	1.14(0.85–1.53)	.391	1.04(0.74–1.47)	.814
Aged 70				
Female (ref. male)	0.72(0.46–1.16)	.176	0.69(0.31–1.55)	.374
BMI < 18.5 (ref. > 18.5)	1.13(0.39–3.32)	.818	0.96(0.32–3.39)	.957
BMI > 25.0 (ref. < 25.0)	2.04(1.21–3.42)	.007	1.90(1.08–3.34)	.026
Living alone (ref. married couple)	0.57(0.26–1.22)	.145	0.48(0.21–1.11)	.086
Married couples(ref. no married couples)	0.58(0.35–0.96)	.034	0.49(0.28–0.86)	.013
Moca-J score	0.96(0.90–1.04)	.302	0.99(0.91–1.08)	.841
Grip strength	1.01(0.98–1.04)	.563	0.99(0.94–1.03)	.581
Serum albumin < 3.8 g/dL(ref. > 3.8 g/dL)	8.67(1.90–39.50)	.005	10.75(1.90–60.73)	.007
Total protein < 6.5 g/dL (ref. > 6.5 g/dL)	2.10(0.42–10.60)	.251	1.39(0.21–9.41)	.737
Carbohydrates > 65%energy(ref. < 65%)	1.27(0.47–3.41)	.640	1.96(0.54–7.21)	.309
Protein < 15%energy (ref. > 15%)	1.90(1.18–3.05)	.008	1.68(0.98–2.88)	.059
Aged 80				
Female (ref. male)	1.67(1.13–2.48)	.010	1.30(0.70–2.44)	.409
BMI < 18.5 (ref. > 18.5)	1.47(0.69–3.14)	.321	1.77(0.79–3.98)	.809
BMI > 25.0 (ref. < 25.0)	0.71(0.42–1.20)	.203	0.76(0.43–1.31)	.323
Living alone (ref. married couple)	1.01(0.62–1.63)	.977	0.83(0.48–1.31)	.516
Married couples(ref. no married couples)	0.90(0.61–1.34)	.605	1.08(0.67–1.73)	.754
Moca-J score	0.96(0.91–1.01)	.089	0.96(0.91–1.02)	.199
Grip strength	0.96(0.93–0.98)	.002	0.97(0.91–1.02)	.186
Serum albumin < 3.8 g/dL(ref. > 3.8 g/dL)	0.58(0.06–4.70)	.581	0.58(0.06–5.30)	.631
Total protein < 6.5 g/dL (ref. > 6.5 g/dL)	2.78(0.55–13.90)	.214	3.10(0.55–17.53)	.286
Carbohydrates > 65%energy(ref. < 65%)	1.82(1.01–3.31)	.040	1.98(0.97–4.06)	.062
Protein < 15%energy (ref. > 15%)	0.94(0.63–1.41)	.765	0.74(0.44–1.21)	.228
Aged 90				
Female (ref. male)	0.89(0.29–2.71)	.831	NA	NA
BMI < 18.5 (ref. > 18.5)	1.94(0.37–10.21)	.434	NA	NA
BMI > 25.0 (ref. < 25.0)	1.85(0.37–9.28)	.455	0.52(0.07–3.76)	.517
Living alone (ref. married couple)	0.40(0.11–1.44)	.162	0.96(0.19–4.94)	.963
Married couples(ref. no married couples)	1.85(0.37–9.28)	.455	6.68(0.57–78.16)	.130
Moca-J score	1.02(0.88–1.19)	.788	1.08(0.87–1.35)	.493
Grip strength	1.05(0.96–1.15)	.307	1.24(1.02–1.51)	.034
Serum albumin < 3.8 g/dL(ref. > 3.8 g/dL)	0.75(0.07–7.69)	.805	NA	NA
Total protein < 6.5 g/dL (ref. > 6.5 g/dL)	NA	NA	NA	NA
Carbohydrates > 65%energy(ref. < 65%)	0.42(0.05–3.91)	.449	NA	NA
Protein < 15%energy (ref. > 15%)	0.14(0.02–1.19)	.072	NA	NA

*BMI* Body Mass Index, *Moca-J* The Japanese version of the Montreal Cognitive Assessment

^a^Univariate logistic regression analysis

^b^Multiple logistic regression analysis evaluated factors of sex, BMI, living alone, married couple, Moca-J score, Grip strength, Serum albumin, Total protein, Carbohydrates energy ratio, and Protein energy ratio

## Discussion

We found that factors associated with weight loss in community-dwelling older people in the present longitudinal study differed among age groups of, 70 and 90 years. Factors associated with weight loss were being baseline over weight (BMI > 25), married couple, low serum albumin levels (< 3.8 g/dL) at age 70, and grip strength at age 90.

These are partly consistent with previous studies showing that being over weight, married couple, grip strength, and low serum albumin levels were factors affecting weight loss among community-dwelling older people [6, 10]. However, unlike our study, these previous studies reported factors affecting weight loss in older adults aged 60 ~ 67 years or older, with an average age of 68.8–73.9 years. In addition, these previous studies reported that different factors associated with weight loss by each age could not be identified. and no significant difference or association was found among current smoking, current drinking, hypertension, DM, cancer and other diseases, blood glucose, HbA1C, calories, and lipid energy ratio in each age group.

The reasons for the different weight loss factors at each age were considered to be the following. In the present study, a small number with weight loss aged 70 were at risk of malnutrition with a low serum albumin level, low protein energy ratio, and low animal protein energy ratio. On the other hand, those aged 70 may have lost weight because they had a higher percentage of BMI over 25 and a higher rate of joint disease than the other age groups. Previous studies showed that the risk of death from weight loss is high for both underweight and overweight individuals 6. In addition, the fact that the risk of weight loss was married-couple supports the findings of previous studies that marital status might be associated with diverse dietary intake and weight loss after losing their spouse [6, 25]. Therefore, it is important to maintain body weight from age around 70.

Our results also suggest that grip strength affected weight loss at age 90. This result differs from previous research showing that weak grip strength affects weight loss [5, 6, 10]. This suggests that the weight loss may have included heavier individuals. However, these studies reported at age 65, and few studies have measured grip strength in those aged 90 in community-dwelling older people. In addition, factors associated with weight loss were not identified at age 80. Those aged 80 may not have been related because of their low serum albumin level, low protein energy ratio, and no high animal protein energy ratio. Weight loss in those aged 70 was influenced by the lifestyle and nutritional status, while weight loss in those aged 90 was influenced by the grip strength, suggesting that weight loss in those aged 80 and 90 may be due to age-related changes. This can also be explained by the fact that the proportion of frail people increases with age [14, 26]. Also, there are five types of frailty trajectories, with the type involved in weight loss focusing on grip strength and the type that progresses to frailty. Two types occur after five years of weight loss, while the present study focused on those after three years [27]. In other words, those aged 80 and 90 may experience weight loss due to age-related changes.

To our knowledge, this is first attempt to specifically study factors associated with weight loss among age groups of, 70 and 90 years in community-dwelling older people in a longitudinal observation. Our finding that factors associated with weight loss among community-dwelling older people differ by age group indicates the need for age-specific preventive interventions.

The strength of this study includes residents from young-old to oldest old, allowing for comparisons by age groups, and targeting older people from diverse living environments in urban and suburban areas, although relatively health-conscious older people were included.

This study had several limitations. Firstly, because we were not able to examine weight changes other than 5% weight loss, we conducted subgroup analysis by 5% weight gain and 10% weight loss, but the number of subjects aged 90 was very small. This may have been reflected in the 5% weight loss due to the lower weight values and small number of subjects at age 90. In addition, there is a selection bias because aged 90 with high physical function participated in the study. This may have been influenced on the results. Furthermore, it is difficult to clarify the causal relationship between weight loss and grip strength due to the small number of subjects. Further research is needed to increase the number of subjects aged 90 and to explain the cause and effect relationship between grip strength and weight loss. Secondly, since BDHQ was used, the amount of physical activity was standardized and did not reflect the amount of physical activity of each subject, so it is necessary to interpret the results carefully. Thirdly, psychological factors such as depression were not assessed. Fourthly, there was no clear distinction between unintentional and intentional weight loss in older people. This may include those with intentional weight loss to improve obesity or diabetes. However, it has been reported that mortality is high even with intended weight loss, and so it is necessary to accumulate knowledge with a clear definition of weight loss [28]. Therefore, our findings need to be confirmed by an intervention study.

In summary, in the current study, we found that factors associated with weight loss by age in community-dwelling older people through a longitudinal study differened by age. Factors associated with weight loss were being over weight, married couple, low serum albumin levels at age 70, and grip strength at age 90. In the future, this study will be useful to propose effective interventions to prevent factors associated with weight loss by age in community-dwelling older people.

## Conclusions

The present study aimed to investigate factors associated with weight loss in community-dwelling older people in a longitudinal observation of different age groups. In the future, this study will be useful to propose effective interventions to prevent factors associated with weight loss by age in community-dwelling older people.

## Data Availability

The datasets used and/or analyzed analyzed during the current study are available from the corresponding author on reasonable request.

## Abbreviations

MoCA-J: Japanese version of the Montreal Cognitive Assessment
SONIC: Septuagenarians, Octogenarians, Nonagenarians, and Investigation with Centenaries
BDHQ: Brief-type self-administered diet history questionnaire
BMI: Body mass index
SD: Standard deviation
CI: Confidence interval
MD: Median
IQR: Interquartile range
